# Comparison of the immunomodulatory potential of platinum-based anti-cancer drugs and anthracyclins on human monocyte-derived cells

**DOI:** 10.1007/s00280-022-04497-1

**Published:** 2022-11-30

**Authors:** Viktória Jenei, Sára Burai, Tamás Molnár, Balázs Kardos, Rebeka Mácsik, Márta Tóth, Zsuzsanna Debreceni, Attila Bácsi, Anett Mázló, Gábor Koncz

**Affiliations:** Department of Immunology, Faculty of Medicine, University of Debrecen, Egyetem Square 1, Debrecen, 4032 Hungary

**Keywords:** Monocyte, Macrophages, Dendritic cells, Cisplatin, Oxaliplatin, Doxorubicin, Epirubicin

## Abstract

Macrophages and dendritic cells (DCs) are important contributors to anti-tumor immune responses. However, these highly plastic cells are also the primary targets of tumor manipulation, which may result in the development of tumor-promoting subtypes. The effect of chemotherapeutic agents on tumor cells is an area of intense study, but little is known about their effects on innate immune cells.

We investigated the effects of four chemotherapeutic drugs (two platinum-based agents; oxaliplatin and cisplatin, and two anthracyclines; doxorubicin and epirubicin) on the differentiation, function, and viability of macrophages and DCs. Macrophages and DCs were differentiated from monocytes in the presence of these chemotherapeutic drugs and we compared their cell surface receptor expression, cytokine production, and chemotactic- and T-cell-polarizing ability.

We have shown that differentiation in the presence of anthracyclines dose-dependently increases CTLA-4 expression in DCs. Antineoplastic agent-driven differentiation strongly modified the CCL2- or CCL5-induced chemotactic activity of both macrophages and DCs. DCs differentiated in the presence of high-dose cisplatin and a low dose of epirubicin promoted regulatory T-cell development, whereas oxaliplatin at specific doses induced both DCs and macrophages to enhance cytotoxic T-cell responses. Furthermore, we found that inflammatory macrophages are more sensitive to doxorubicin-induced cell death than their counterparts.

In summary, our results confirm that chemotherapeutic agents acting on a similar basis may have different effects on the anti-tumor immune response. Treatment with optimal dose, combinations, and timing of chemotherapy may determine tumor immunity and the metastatic potential of tumors.

**Supplementary Information:**

The online version contains supplementary material available at 10.1007/s00280-022-04497-1.

## Introduction

Macrophages and dendritic cells (DCs) play a critical role in tissue homeostasis and are involved in a number of pathophysiological conditions, including cancer. Both infiltrating and tissue-resident forms of these highly plastic cells adapt to the conditions of the tumor microenvironment (TME). Macrophages and DCs can influence the immunological microenvironment, tumor growth, the induction and maintenance of cancer stem cells, epithelial-to-mesenchymal transition (EMT), as well as dissemination and metastasis of tumor cells [1]. In addition, macrophages, and DCs initiate and modulate the adaptive immune responses, through which these cells regulate cancer progression either by controlling effector T lymphocytes or by enhancing the functions of regulatory T cells [2].

The importance of optimizing the concentration, timing, and combination of chemotherapeutic agents has become apparent in improving therapeutic responses [3]. Current clinical practice in chemotherapy aims to achieve a systemic drug concentration, based on patient-specific body measurements (e.g., body surface area (BSA) [3]. However, not only cancer cells but also macrophages and DCs, as well as their precursors, are exposed to the agents used, which can significantly affect the success of a therapeutic intervention. To win the fight against cancer, not only strategies need to be developed to effectively kill cancer cells, but also attempts must be made to stimulate the immune system so that it can control the residual tumor cells [4]. The conditions of the developmental and differentiation process are crucial for the subsequent role of both macrophages and DCs. Future therapeutics should consider the effects of the treatments used, such as chemotherapies, on the differentiation and functions of these highly plastic cells. In our study, we investigated two platinum-based antineoplastic agents; oxaliplatin and cisplatin, with the ability to crosslink with the urine bases on the DNA leading to DNA damage and subsequently apoptosis within cancer cells and two anthracyclines; doxorubicin and epirubicin with DNA intercalation ability and consequently inhibition of nucleic acid and protein synthesis resulting in cytocidal activity.

As a possible therapeutic approach, reprogramming of macrophage or DC differentiation is an intensively studied area, but the effects of currently applied anti-cancer therapies on innate immune cell differentiation have been hardly studied. Suppression or modulation of the immune response with improperly applied conventional chemotherapy may contribute to the development of immune escape mechanisms of tumors, but optimizing the use of chemotherapeutic agents can also have a positive effect on the immune status of the tumor microenvironment [5, 6]. We explored the effects of four conventional chemotherapeutic drugs on the differentiation of human monocyte-derived macrophages and DCs. We investigated cell surface marker expression, cytokine production, chemotactic- and T-cell polarization ability, and cell death sensitivity of macrophages and DCs differentiated in the presence of each agent.

## Materials and methods

### Differentiation of human macrophages and DCs

Heparinized leukocyte-enriched buffy coats were obtained from healthy blood donors drawn at the Regional Blood Center of the Hungarian National Blood Transfusion Service (Debrecen, Hungary) in accordance with the written approval of the Director of the National Blood Transfusion Service and the Regional and Institutional Research Ethical Committee of the University of Debrecen, Faculty of Medicine (Debrecen, Hungary). Written, informed consent was obtained from the blood donors prior to blood donation, their data were processed and stored according to the directives of the European Union. Peripheral blood mononuclear cells (PBMCs) were separated from buffy coats by Ficoll-Paque Plus (Amersham Biosciences) gradient centrifugation. According to the manufacturer, monocytes were purified from PBMCs by positive selection using immunomagnetic cell separation and anti-CD14-conjugated microbeads (Miltenyi Biotec) instruction. After separation on a VarioMACS magnet, 96–99% of the cells were shown to be CD14^+^ monocytes, as measured by flow cytometry. Isolated monocytes were plated at 1.5 × 10^6^ cell/ml concentration in Gibco’s serum-free AIM-V medium (Thermo Fischer Scientific). To acquire the DCs or macrophages, 100 ng/ml IL-4 (PeproTech EC) and 80 ng/ml GM-CSF (Gentaur Molecular Products) or 50 ng/ml M-CSF (PeproTech) were added to the cells on the day of separation. Half the volume of medium supplemented with 100 ng/ml IL-4 and 80 ng/ml GM-CSF or 50 ng/ml M-CSF depending on the cell type was updated on day 2 of the differentiation process. Chemotherapeutic agents, Cisplatin (APExBIO), Oxaliplatin APExBIO), Doxorubicin (Teva), and Epirubicin (Teva) were added to the cell culture immediately following isolation of the monocyte, and on the second day of the differentiation process.

### Measurement of cell death intensity of macrophage and dendritic cell subpopulations

M1 and M2 macrophages were generated as we described [7]. Shortly, monocytes differentiated in the presence of M-CSF were stimulated on the fifth day of differentiation for 24 h with lipopolysaccharide (50 ng/ml ultrapure LPS, InvivoGen), IFNγ (20 ng/ml, PeproTech) to obtain M1 and IL-4 (20 ng/ml, PeproTech), IL-10 (20 ng/ ml, PeproTech) and TGFß (20 ng/ml, PeproTech) to get M2 phenotype. As widely accepted, dexamethasone turns tumor antigen-presenting cells into tolerogenic dendritic cells with T-cell inhibitory functions [8, 9]. To examine the effect of chemotherapeutic agents on the viability of tolerogenic DCs, dexamethasone DCs (dexDCs) were generated as we described [10]. Briefly, isolated monocytes were cultured with 100 ng/ml IL-4, 0.25 µM dexamethasone (Sigma-Aldrich), and 80 ng/ml GM-CSF for 5 days. Differentiated cells were treated with chemotherapeutic agents for 24 h. Cell death was determined based on propidium iodide uptake (PI, Sigma-Aldrich). PI (10 µg/ml) was added to the cells directly before analysis by flow cytometry. Cell death was measured by flow cytometry using FACS Calibur (BD Biosciences), and data were analyzed by FlowJo v X.0.7 software (Tree Star).

### Detection of cell surface markers

Phenotyping of conditioned monocyte-derived DCs (moDCs) and macrophages was performed by flow cytometry, anti-human CD14-fluorescein isothiocyanate (FITC), CD209/DC-SIGN-phycoerythrin (PE), CD1a-FITC, CD80-FITC CD86-PE, PD-L1-PE, CTLA-4-PE, CD163-PE, CD206-PE (BioLegend), HLA-DQ-FITC (BD Biosciences) were used.

### Determination of cell viability

Total cell death was quantified based on the loss of membrane integrity and the uptake of propidium iodide (PI, Sigma-Aldrich) or 7AAD(Invitrogen). Cells were stained with PI (10 μg/ml)) or by (0.25 μg/ml) 7AAD before analysis by flow cytometry. Cell death was measured by flow cytometry using FACS Calibur (BD Biosciences), and data were analyzed by FlowJo software (Tree Star). Additionally, cell viability was measured with Cell Counting Kit-8 (ApexBio; #K1018) according to the manufacturer’s protocol. Briefly, 10 μL CCK-8 solution was added to each well and then incubated for 4 h. Absorbance was measured with EnVision® 2105 multimode plate reader (PerkinElmer, Waltham, MA, USA) at 450 nm. All experiments were performed in quadruplicate, and cell viability (%) was expressed as a percentage relative to the untreated control cells.

### Measurement of the cytokine and chemokine concentrations

Culture supernatants from moDCs and macrophages were harvested 5 days after monocyte separation and the concentration of IL-10, TGFβ, IFNγ, IL-12, IL-1β, and TNFα cytokines, as well as IP-10 and IL-8 chemokines, was measured and validated by using OptEIA kits (BD Biosciences) following the manufacturer’s instructions.

### Migration assay

According to the Boyden Chamber Assay Protocol, 5 × 10^4^ cells were added to the upper chamber, and the migration of DCs and macrophages was measured in the presence or absence of 3 ng/ml CCL2 or 10 ng/ml CCL5 chemokines placed in the lower chamber. After 4 h of incubation at 37°, the migrated cells from the lower chamber were collected. Migrated cells then were quantified by counting the cells by Novocyte2000R Flow Cytometer (Agilent (Acea) Biosciences Inc., USA), and data were analyzed by the FlowJo v X.0.7 software (Tree Star).

### Detection of T-lymphocyte polarization

Monocyte-derived DCs and macrophages were counted, washed, and then co-cultured with allogeneic peripheral blood lymphocytes (PBL) for three, five, or nine days in RPMI-1640 medium (Sigma-Aldrich) at a moDC/macrophage: T-cell ratio of 1: 10 at 37 °C. To determine which T-lymphocyte populations were polarized by the preconditioned DCs and macrophages after three, five or nine days the T cells were stimulated with 1 µg/ml ionomycin and 20 ng/ml phorbol-myristic acetate (PMA) for 4 h, and the vesicular transport was inhibited by BD GolgiStop™ protein transport inhibitor (BD Biosciences) 12 h before the cell staining. The cells were labeled with anti-human CD4-Peridinin Chlorophyll Protein Complex (PerCP), CD8-PE and/or anti-human CD25-PE-conjugated antibodies (BioLegend). Following this, they were fixed and permeabilized by using BD Cytofix/Cytoperm™ Plus Fixation/Permeabilization Kit (BD Biosciences) and labeled with anti-human IFNγ-APC (BD Biosciences), anti-human IL-4-PE (R&D Systems), anti-human IL-10-Alexa Fluor 488, anti-human IL-17-PE (BioLegend), and anti-human FoxP3-APC (R&D System) antibodies. Fluorescence intensities were measured by FACS Calibur cytometer (BD Biosciences) and data were analyzed by the FlowJo v X.0.7 software (Tree Star).

### Measurement of ROS production

Macrophages or DCs were stimulated with 100 nM phorbol 12‐myristate 13‐acetate (PMA). The cellular ROS were measured by 2’,7’ -dichlorofluorescein diacetate (DCF-DA) assay kit (Invitrogen™ and Carboxy-H2DCFDA C400). The cells were plated on 96-well black-wall/clear bottom plates. They were rinsed twice in PBS and treated with 50 µM DCF-DA (freshly diluted in PBS) for 15, 30 min and 1, 1.5, 2, 3, 4, 5, and 6 h at 37 ^◦^C with the light blocked. Following incubation times, fluorescence signals were detected using a microplate reader.

### Statistical analysis

Analyses were performed using Excel (Microsoft Corporation) and GraphPad Prism Version 6.0 (GraphPad Software Inc.) software. Differences were considered to be statistically significant at *P* < 0.05. In the statistical analysis, ANOVA followed by Bonferroni’s post hoc test was used for the comparison. The results were expressed as mean ± standard deviation. Differences were considered to be statistically significant at *p* < 0.05. Significance was indicated as ^#^*p* < 0.05, ^##^*p* < 0.01 and ^###^*p* < 0.001 compared to untreated control cells and as *p < 0.05, **p < 0.01 and ***p < 0.001 compared to treated counterparts.

## Results

### Doxorubicin and epirubicin enhance the expression of cell surface markers characteristic of tolerogenic cell subpopulations

We investigated the effects of four chemotherapeutic agents on the differentiation of macrophages and DCs. To test this, blood-derived CD14^+^ monocytes were differentiated into macrophages and DCs in the presence of M-CSF or GM-CSF + IL-4 for five days as previously published [7, 10]. Immediately after isolation of the monocytes, chemotherapeutic agents were added to the cell culture and these drugs were present in the culture for all five days. In clinical application, the maximum plasma concentration of the drugs used at the recommended highest dose was in the range of 4–16 µM [11]. The dose range used in our in vitro experiments for cisplatin and oxaliplatin corresponds to these therapeutic concentration range. For doxorubicin and epirubicin, we used approximately one-tenth of these doses, that were not yet toxic during monocyte differentiation. The toxicity of the applied concentration ranges to monocyte-derived cells was checked by 7AAD staining and CCK assay (SFigure 1 A and B).

**Fig. 1:**
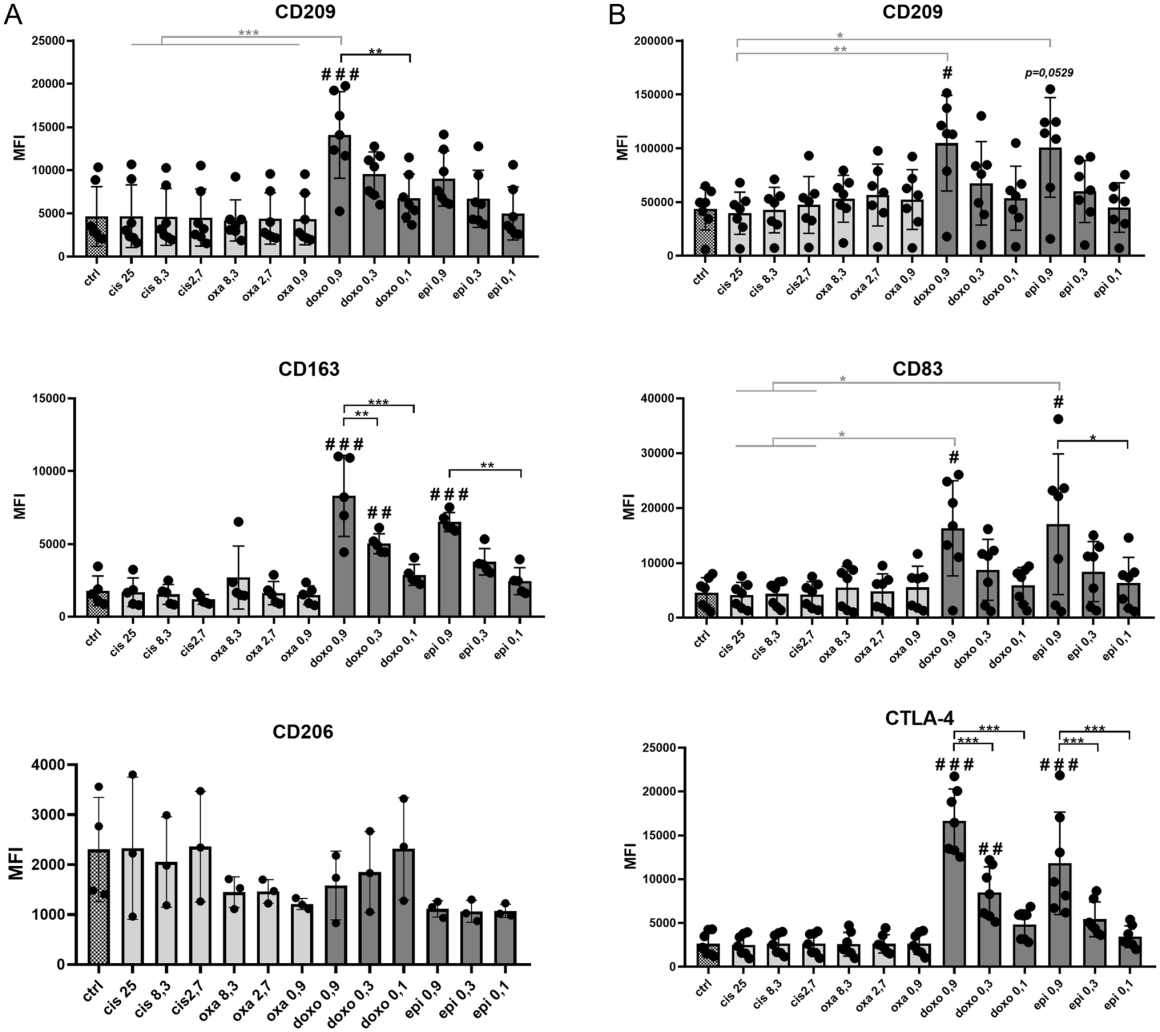
The presence of chemotherapeutic agents during differentiation modifies the pattern of cell surface markers in macrophages and dendritic cells. CD14^+^ monocytes were cultured with recombinant 50 ng/ml recombinant M-CSF (for macrophages) or 100 ng/ml IL-4 and 80 ng/ml GM-CSF (for dendritic cells). Cisplatin/oxaliplatin/doxorubicin/epirubicin was added to freshly isolated monocytes at the indicated doses for five days. On day five, the cell surface expression of **A** CD209, CD163, and CD206 were analyzed on monocyte-derived macrophages and **B** CD209, CD83 and CTLA-4 was detected on monocyte-derived dendritic cells by flow cytometry. The MFI (median fluorescence intensity) and the mean values of the cells' ratio positive for the measured surface molecules were calculated from five independent experiments + SD at least. In the statistical analysis, ANOVA followed by Bonferroni’s post hoc test was used for the comparison. The results were expressed as mean ± standard deviation. Differences were considered to be statistically significant at *p* < 0.05. Significance was indicated as #*p* < 0.05, ##*p* < 0.01 and ###*p* < 0.001 compared to untreated control cells and as *p < 0.05, **p < 0.01 and ***p < 0.001 compared to treated counterparts

First, we compared the expression of cell surface molecules in the presence or absence of the four chemotherapeutic agents (Fig. 1). During the cell characterization the expression of CD209, CD163, CD206 (Fig. 1A), HLA-DQ, CD14, CD86, CTLA-4 and PD-L1 (SFigure 2) was measured on the surface of macrophages, while in the case of DCs the expression of CD209,, CD83, CTLA-4 (Fig. 1 B), CD206, CD80, CD1a and PD-L1 (SFigure 3) was monitored by flow cytometry. Significant changes were identified neither in the case of CD14, CD206, CD86, CTLA-4 and PD-L1 markers on macrophages nor in the case of CD206, CD80 and PD-L1 molecules on the surface of DCs (SFigures 2 and 3). Cisplatin and oxaliplatin treatments did not alter significantly the expression of any of the characteristic molecules on either macrophage (Fig. 1A) or DCs (Fig. 1B) compared to the control sample. In contrast, the presence of doxorubicin and epirubicin dose-dependently increased the expression of CD209 and CD163 markers, characteristics of the anti-inflammatory M2 macrophage subpopulation. Meanwhile, these treatments did not alter the expression of CD206, another prominent M2 marker, and did not reduce the expression of the M1 marker, CD14 (Fig. 1A and SFigure 2). Expression of the C-type lectin mannose receptor, CD209 (DC-SIGN), the maturation marker CD83, and the CTLA-4 costimulatory molecule was dose-dependently increased in DCs after doxorubicin and epirubicin treatments (Fig. 1B).

In summary, in the presence of doxorubicin and epirubicin, the differentiation of macrophages and DCs from monocytes is altered, resulting in the development of unique, presumably tolerogenic phenotypes.

### Anthracyclines and platinums modify cytokine production of monocyte-derived cells

**Fig. 2:**
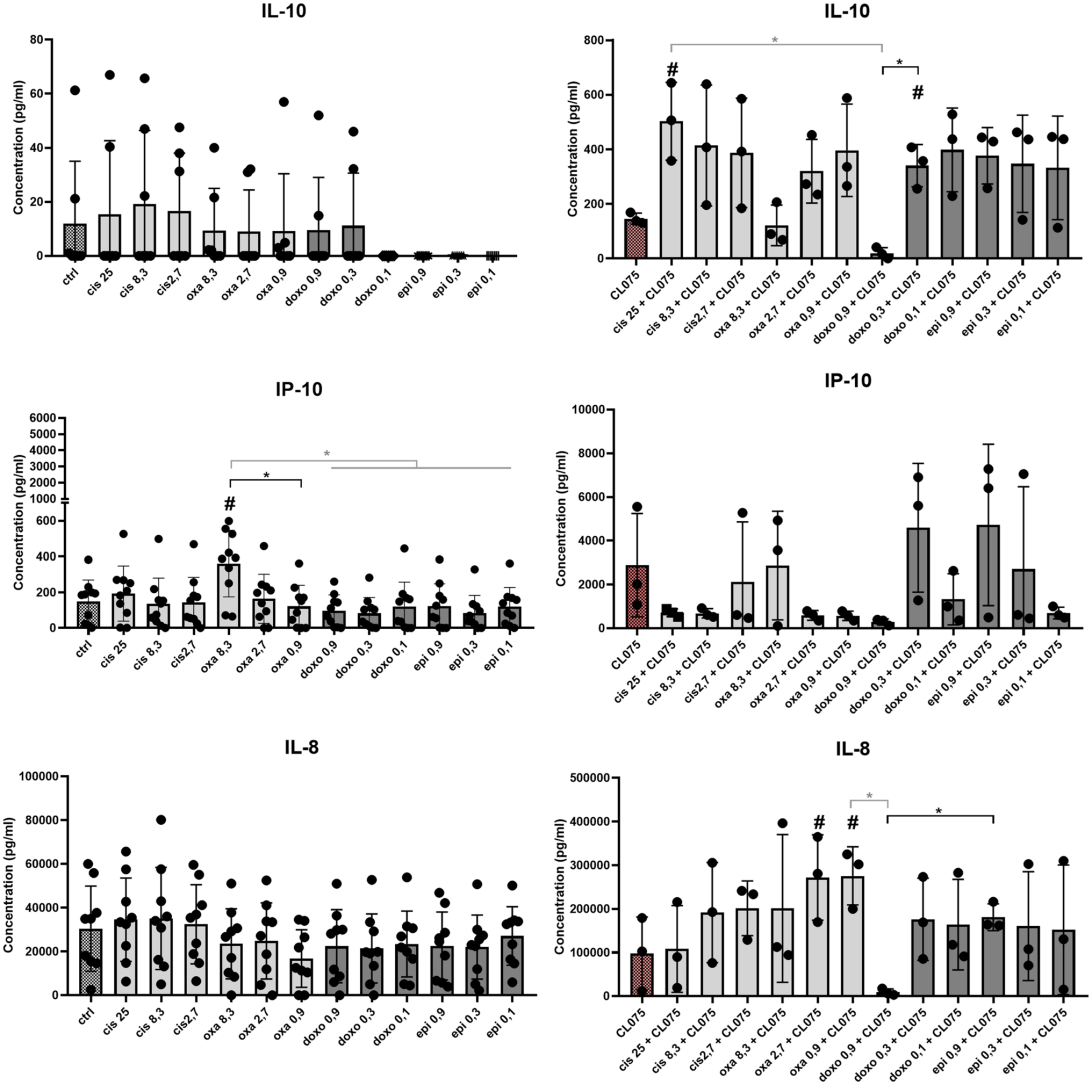
Chemotherapeutic agent-driven differentiation modulates the cytokine and chemokine production of macrophages. CD14^+^ monocytes were cultured with 50 ng/ml recombinant M-CSF (for macrophages) in the presence of cisplatin/oxaliplatin/doxorubicin or epirubicin for five days. The concentration of secreted IL-10, IP-10, IL-8 production of macrophageswas detected by ELISA on day five of differentiation. On day 5 of in vitro macrophage differentiation the cells were activated by 1 μg/ml CL-075 for 24 h obtained from InvivoGen (Toulouse, France). In the statistical analysis, ANOVA followed by Bonferroni’s post hoc test was used for the comparison. The results were expressed as mean ± standard deviation. Differences were considered to be statistically significant at *p* < 0.05. Significance was indicated as #*p* < 0.05 compared to untreated control cells and as *p < 0.05 compared to the chemotherapeutic agent-treated counterparts

**Fig. 3:**
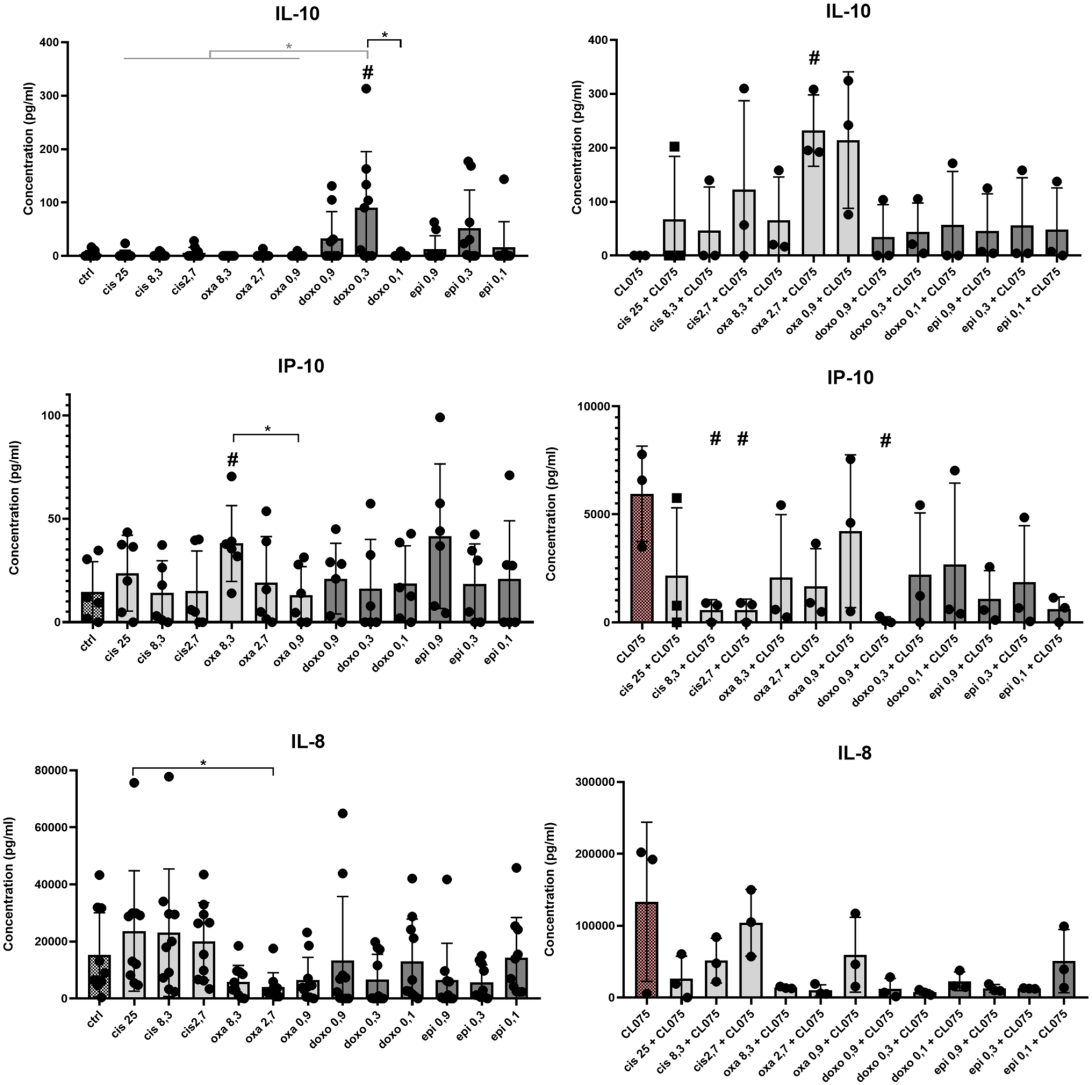
Chemotherapeutic agent-driven differentiation modulates the cytokine and chemokine production of dendritic cells. CD14^+^ monocytes were cultured with 100 ng/ml IL-4 and 80 ng/ml GM-CSF in the presence of cisplatin/oxaliplatin/doxorubicin or epirubicin for five days. The concentration of secreted IL-10, IP-10, IL-8 production of dendritic cells was detected by ELISA on day five of differentiation. On day 5 of in vitro DC differentiation the cells were activated by 1 μg/ml CL-075 for 24 h obtained from InvivoGen (Toulouse, France). In the statistical analysis, ANOVA followed by Bonferroni’s post hoc test was used for the comparison. The results were expressed as mean ± standard deviation. Differences were considered to be statistically significant at *p* < 0.05. Significance was indicated as #*p* < 0.05 compared to untreated control cells and as *p < 0.05 compared to the chemotherapeutic agent-treated counterparts

To test the functionality of monocyte-derived cells, we examined the cytokine profile of macrophages and DCs differentiated in the presence of chemotherapeutic agents (Figs. 2 and 3). High concentration (8.3 µM) of oxaliplatin significantly increased the IP-10 chemokine secretion by macrophages, whereas the other platinum, cisplatin treatment proved to be ineffective (Fig. 2). Similar to macrophages, 8.3 µM)of oxaliplatin treatment increased IP-10 secretion by DCs. However, in contrast to macrophages, increased IL-10 production was observed in the case of DCs differentiated in the presence of 0.3 µM doxorubicin (Fig. 3). Either macrophages or DCs were examined, we could not detect any differences in the production of IFNγ, IL-1β, IL-6 TNFα, or IL-12 between untreated cells and cells differentiated in the presence of the chemotherapeutic drugs *(data not shown)*. We also found no differences in ROS production of macrophages and DCs differentiated under the influence of anti-cancer drugs (SFigure 4).

To test how cells cultured with or without anti-cancer drugs respond to activation stimuli, we stimulated cells with the TLR8 agonist CL-075, which is both effective at activating macrophages and DCs in serum-free media as well. In response to CL-075 activation, macrophages differentiated in the presence of 25 µM cisplatin produced higher amounts of tolerogenic IL-10, while macrophages differentiated in the presence of lower doses of oxaliplatin produced higher amounts of the chemokine IL-8 than their counterparts differentiated under normal conditions (Fig. 2). Oxaliplatin treatment (2.7 µM) in DCs resulted in elevated IL-10 production, while the presence of a lower dose of cisplatin significantly reduced the IP-10 secretion following CL-075 stimulation (Fig. 3).

Our results indicate that there are differences in the effect of doxorubicin/epirubicin or oxaliplatin/cisplatin on chemokine production, and the different behavior of oxaliplatin/cisplatin can also be observed in the presence of activation signals. We also have shown that the effect of chemotherapeutic agents on cytokine production may differ in a cell type-dependent manner.

### There are intratypic differences in the effect of anthracyclines and platinums on the chemotactic ability of monocyte-derived cells

The ability of chemotherapy to influence metastasis has become evident in cancer therapy. The development of tumor metastasis and the pre-metastatic gap, the so-called invasion-metastasis cascade, involves several consecutive and interrelated biochemical and immunological events [12]. Chemokine ligand 2 (CCL2), also known as monocyte chemotactic protein 1 (MCP-1), has a strong chemotactic ability to recruit monocytes and macrophages to TME [6]. In addition, CCL5, also known as RANTES (activated and regulated by normal T-cell expression and secretion), also strongly promotes carcinogenesis and stromal development [13]. Therefore, we wanted to investigate how the presence of chemotherapeutic agents during monocyte differentiation affects the ability of macrophages and DCs to migrate to CCL2 or CCL5 chemokines using a semipermeable membrane cell culture insert.

**Fig. 4:**
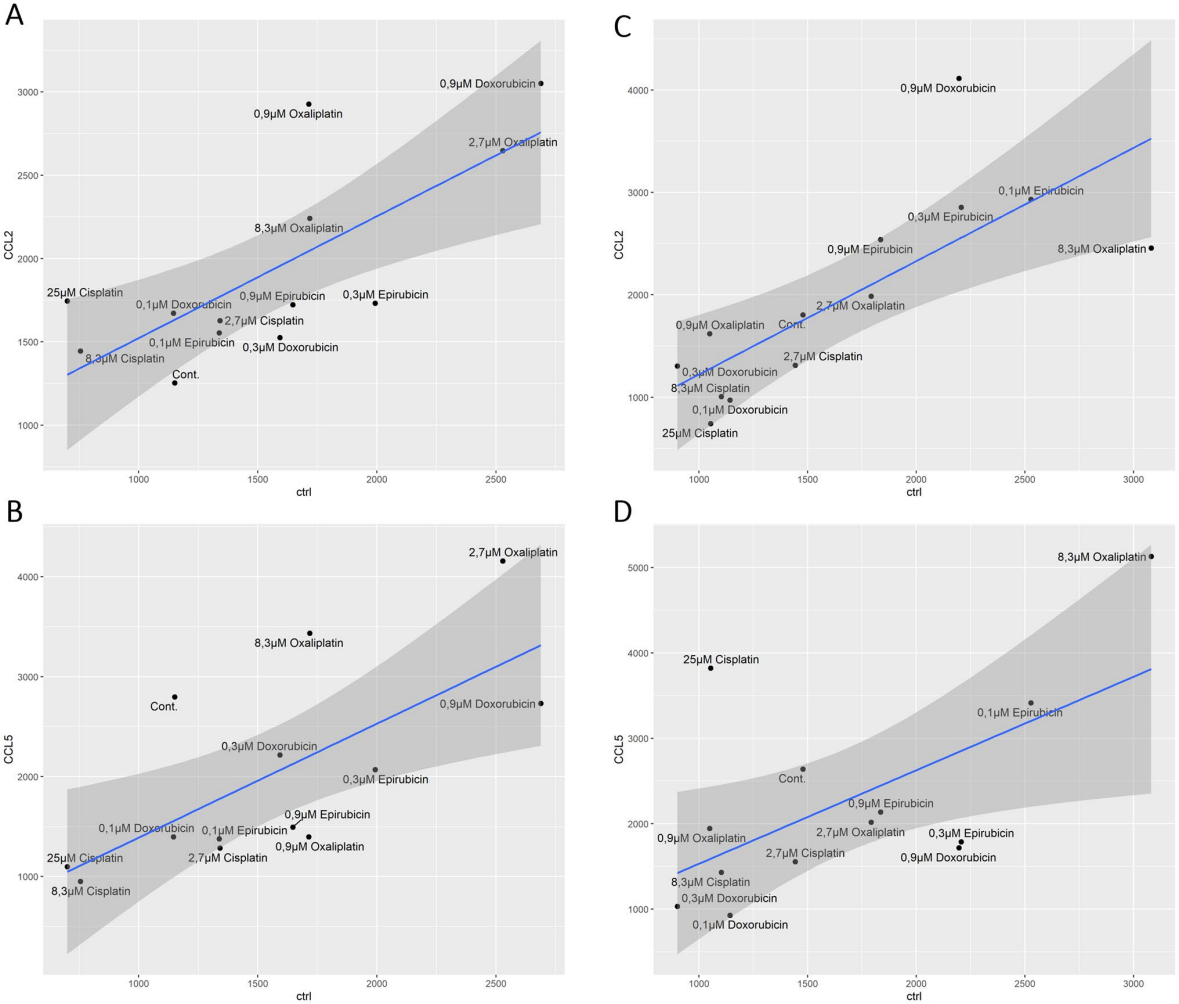
The presence of chemotherapeutic agents during differentiation modifies the chemotactic ability of macrophages and dendritic cells toward CCL2 and CCL5 chemokines. CD14^+^ monocytes were cultured with 50 ng/ml recombinant M-CSF (for macrophages) or 100 ng/ml IL-4 and 80 ng/ml GM-CSF (for dendritic cells) in the presence of cisplatin/oxaliplatin/doxorubicin or epirubicin for five days. On day five, the migration intensity of the macrophages and dendritic cells was determined according to cell number using a semipermeable membrane cell culture insert. The scatter plots show the motility of control and chemotherapeutics-conditioned **(A and B)** macrophages and **(C and D)** dendritic cells induced by 10 ng/ml CCL2 **(A and C)** or 3 ng/ml CCL5 **(B and D)** compared to those control samples without chemokines. The number of migrated cells was determined from the average of three independent experiments. Scatter plots were made by GGplot2 in the R studio platform

First, we compared the effects of chemotherapeutic agents on the migration capacity of cells without chemokine treatment. (The change in migration activity compared to non-treated cells is shown by the shift on the x axis of the Fig. 4). In the presence of oxaliplatin, macrophages showed overall elevated migration potential, while a higher doses of cisplatin (8.3 μM and 25 μM) resulted in reduced migratory capacity of macrophages. Differentiation in the presence of doxorubicin and epirubicin dose-dependently increased the migration capacity of macrophages (Fig. 4A and B). During cisplatin-modified differentiation, the migratory potential of DCs decreased, whereas the presence of oxaliplatin during differentiation increased it in a dose-dependent manner. Epirubicin treatment during differentiation, in contrast to lower doses of doxorubicin treatment (0.1 μM and 0.3 μM) increased the migration capacity of the developed DCs (Fig. 4C and D).

After that, we compared the effects of chemotherapeutic agents on the migration capacity of cells in the presence of CCL2 or CCL5 chemokines (chemokine-modified migration is shown by its shift on the y-axis of Fig. 4). The presence of oxaliplatin and a high dose of doxorubicin (0.9 μM) during differentiation increased macrophage chemotaxis to CCL2 (Fig. 4A). The movement of macrophages to CCL5 was reduced by cisplatin-driven, but increased by the higher dose of oxaliplatin-driven differentiation. Epirubicin and lower doses of doxorubicin-driven differentiation resulted in the macrophage population being less prone to move toward CCL5 (Fig. 4B).

During cisplatin-modified differentiation, the migratory potential of DCs decreased migration toward CCL2 (Fig. 4C). The addition of a relatively high dose of cisplatin (25 μM) or oxaliplatin (8.3 μM) during monocyte differentiation increased its intensity toward CCL5 (Fig. 4D). The presence of high-dose doxorubicin (0.9 μM) increased DC movement to CCL2 (Fig. 4C) but decreased to CCL5 (Fig. 4D). In contrast, epirubicin-modulated differentiation did not alter the movement of DCs toward CCL2 or CCL5 (Fig. 4C and D).

In summary, the presence of different chemotherapeutic drugs during the differentiation of monocytes individually modified the migration potential of macrophages and DCs and the chemotaxis of these cells to CCL2 and CCL5.

### Chemotherapeutic agents-conditioned macrophages and DCs tend to polarize T cells into regulatory T cell or IFNγ-producing T-cell direction

**Fig. 5:**
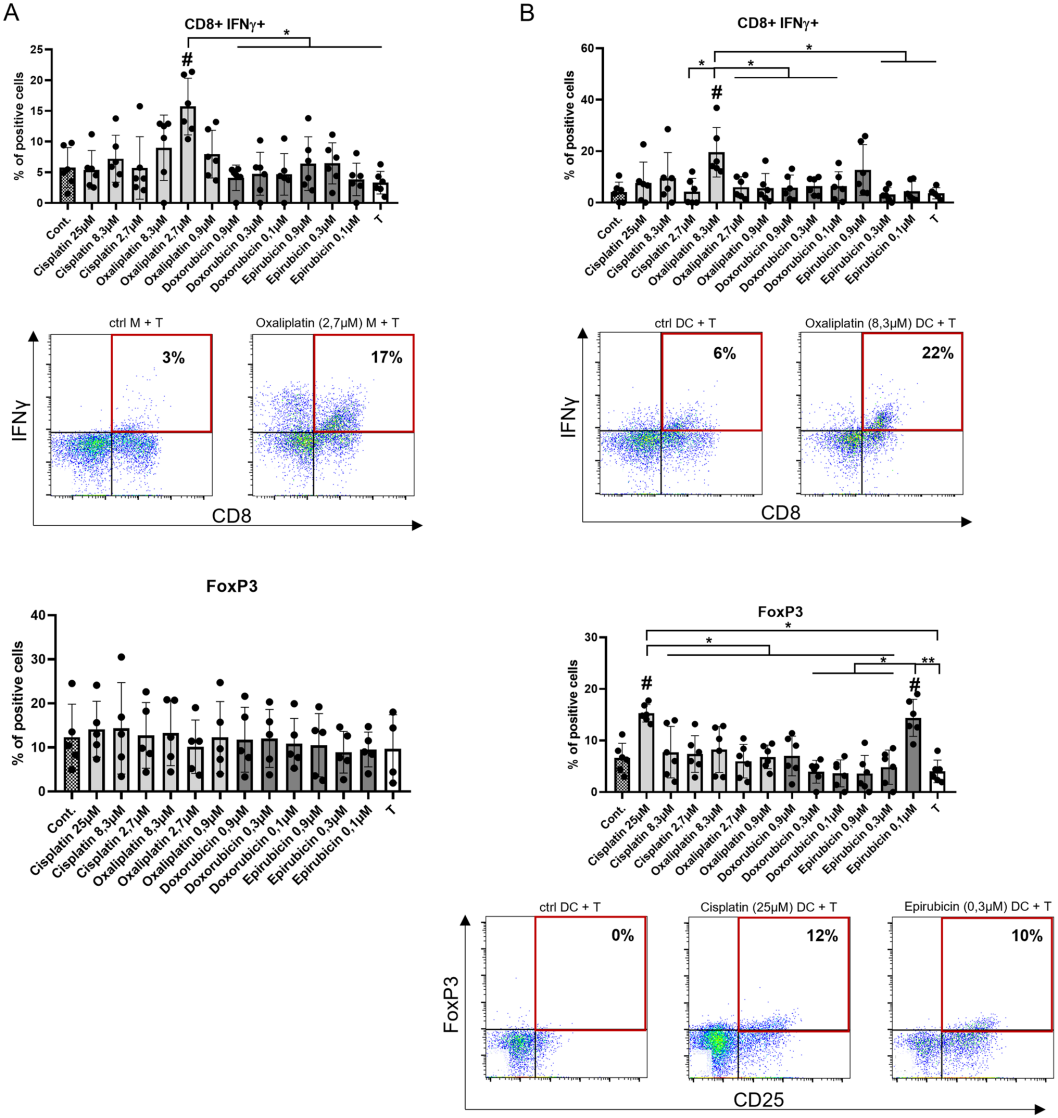
Chemotherapeutic agent-driven differentiation differently alter the T-cell-polarizing capacity of macrophages and dendritic cells. CD14^+^ monocytes were cultured with 50 ng/ml recombinant M-CSF (for macrophages) or 100 ng/ml IL-4 and 80 ng/ml GM-CSF (for dendritic cells) in the presence of cisplatin/oxaliplatin/doxorubicin or epirubicin for five days. On day five macrophages or dendritic cells were co-cultured with allogenous peripheral blood lymphocytes (PBL) at a monocyte-derived cell: T-cell ratio of 1: 10 at 37 °C.”T” sample indicates control consisting of only T cells without monocyte-derived cells. After three (CD8^+^ Tc) or nine days (CD4^+^ Treg) the T cells were stimulated with 1 μg/ml ionomycin and 20 ng/ml phorbol-myristic acetate (PMA) for 4 h, and the vesicular transport was inhibited. The ratio IFNγ producing cytotoxic T cells and CD4^+^ regulatory T lymphocytes (CD25^+^ IL-10^+^) were detected after the co-culturing of PBL with control or chemotherapeutics-conditioned macrophages (**A**) or dendritic cells (**B**). Representative dot plots of samples showing significant differences are shown. The cells' ratio for the measured molecules was calculated from at least five independent experiments + SD and In the statistical analysis, ANOVA followed by Bonferroni’s post hoc test was used for the comparison. The results were expressed as mean ± standard deviation. Differences were considered to be statistically significant at *p* < 0.05. Significance was indicated as #*p* < 0.05 compared to untreated control cells and as *p < 0.05 compared to the chemotherapeutic agent-treated counterparts

We co-cultured macrophages and DCs with T cells to explore the T-cell-polarizing ability of these monocyte-derived cells (Fig. 5). To characterize T-cell differentiation, in addition to CD4 and CD8 markers, we also checked the expression of CD25, the intracellular level of FoxP3 and IL-10 cytokine as regulatory T-cell markers, and intracellular expression of IL-4, IL-17, and IFNγ as markers of Th2, Th17 and Th1 cells, respectively. (The gating strategy is presented in SFigure 5.) Definite doses of oxaliplatin modulated the differentiation of both DCs and macrophages, resulting in an increase in the proportion of INF gamma-positive cytotoxic T cells (Figure A and B). An increase in the proportion of FoxP3-positive T cells was observed when T cells were co-cultured with DCs differentiated in the presence of high-dose cisplatin or low-dose epirubicin (Fig. 5B). The presence of any of the chemotherapeutic agents during the differentiation of monocyte-derived macrophages or DCs did not modify the polarization of co-cultured T cells into IL-4- and IL-17-producing subtypes *(data not shown)*. Based on our results, the effect of the two platinums on monocyte differentiation is significantly different in terms of the T-cell polarizing ability of the formed DCs; oxaliplatin increased the level of cytotoxic T cells, while cisplatin increased the number of regulatory T cells.

### Inflammatory macrophage subpopulation is more sensitive to chemotherapy-induced cell death than their counterpart

**Fig. 6:**
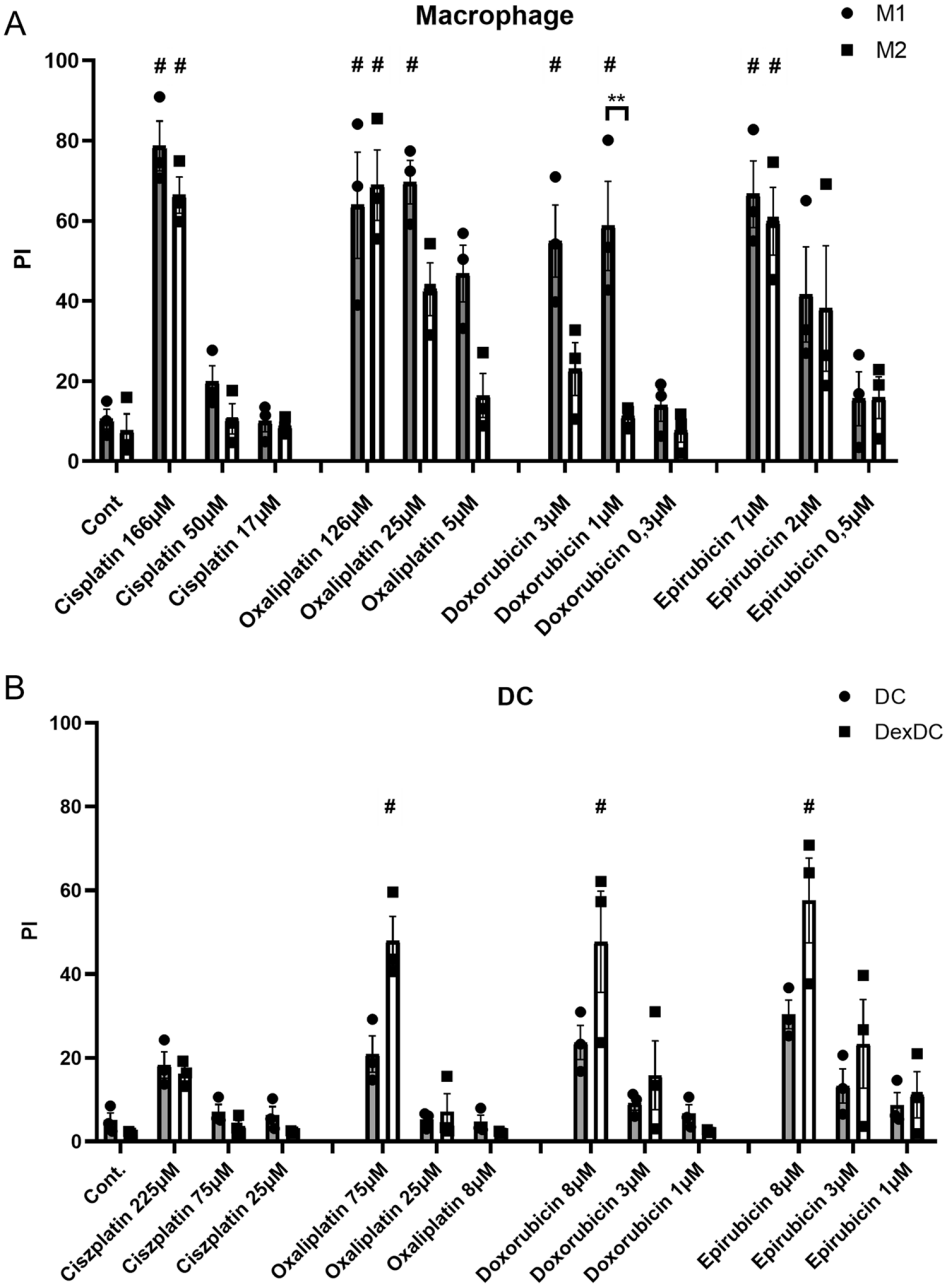
Inflammatory and tolerogenic macrophages and dendritic cells differ in susceptibility to cell death treated with chemotherapeutic agents **(A)** CD14^+^ monocytes were cultured with 50 ng/ml recombinant M-CSF for five days, monocyte-derived macrophages were differentiated to M1 and M2 phenotype in the presence of LPS (50 ng/ml) and IFNγ (20 ng/ml) or IL-4 (20 ng/ml), IL-10 (20 ng/ml) and TGFß (20 ng/ml), respectively **(B)**. For dendritic cells differentiation, CD14^+^ monocytes were cultured with recombinant 100 ng/ml IL-4 and 80 ng/ml GM-CSF with or without 0.25 µM dexamethasone for five days. The in vitro-differentiated human M1 and M2 cells or dendritic cells and dexamethasone-treated dendritic cells (DexDCs) were treated with the indicated doses of cisplatin/oxaliplatin/doxorubicin or epirubicin on day five for 24 h. After 24 h, cell death was determined by PI staining. The ratio of cell death was calculated from four independent experiments + SD. In the statistical analysis, ANOVA followed by Bonferroni’s post hoc test was used for the comparison. The results were expressed as mean ± standard deviation. Differences were considered to be statistically significant at *p* < 0.05. Significance was indicated as #*p* < 0.05 compared to untreated control cells and as **p < 0.01 between the M1 and M2 cells

The balance of pro-inflammatory or anti-inflammatory cell populations can be regulated not only by directing their differentiation but also by harnessing their different sensitivity to cell death. Macrophages were differentiated into M1 and M2 subtypes using LPS and IFNγ or IL-4, IL-10 and TGFβ. Classical and tolerogenic DC differentiation was induced, the latter, as published, using dexamethasone [10]. Fully differentiated macrophages and DC subpopulations were treated with different doses of the four antineoplastic drugs. Effective killing required doses approximately tenfold higher than those used for monocyte differentiation under the experimental conditions described above. M1 macrophages were typically more sensitive to chemotherapy-induced cell death than M2 macrophages. This difference was most pronounced for doxorubicin; a significant difference was detectable between M1’ and M2’ cell death sensitivity upon 1 μM doxorubicin treatment. Interestingly, the addition of the other anthracycline, epirubicin, did not induce more intense cell death in M1 than in M2 macrophages (Fig. 6A). DCs were sensitive to cell death induced by oxaliplatin, doxorubicin, and epirubicin, but both DC subpopulations were resistant to cisplatin-induced cell death (Fig. 6B). Typically, tolerogenic DC subtypes were more sensitive to chemotherapy-induced cell death than their counterparts, but these differences were not significant. Based on our results, the use of high-dose chemotherapy may be suitable for influencing the balance of tolerogenic/immunogenic macrophages and DC subpopulations in the tumor microenvironment.

## Discussion

**Fig. 7:**
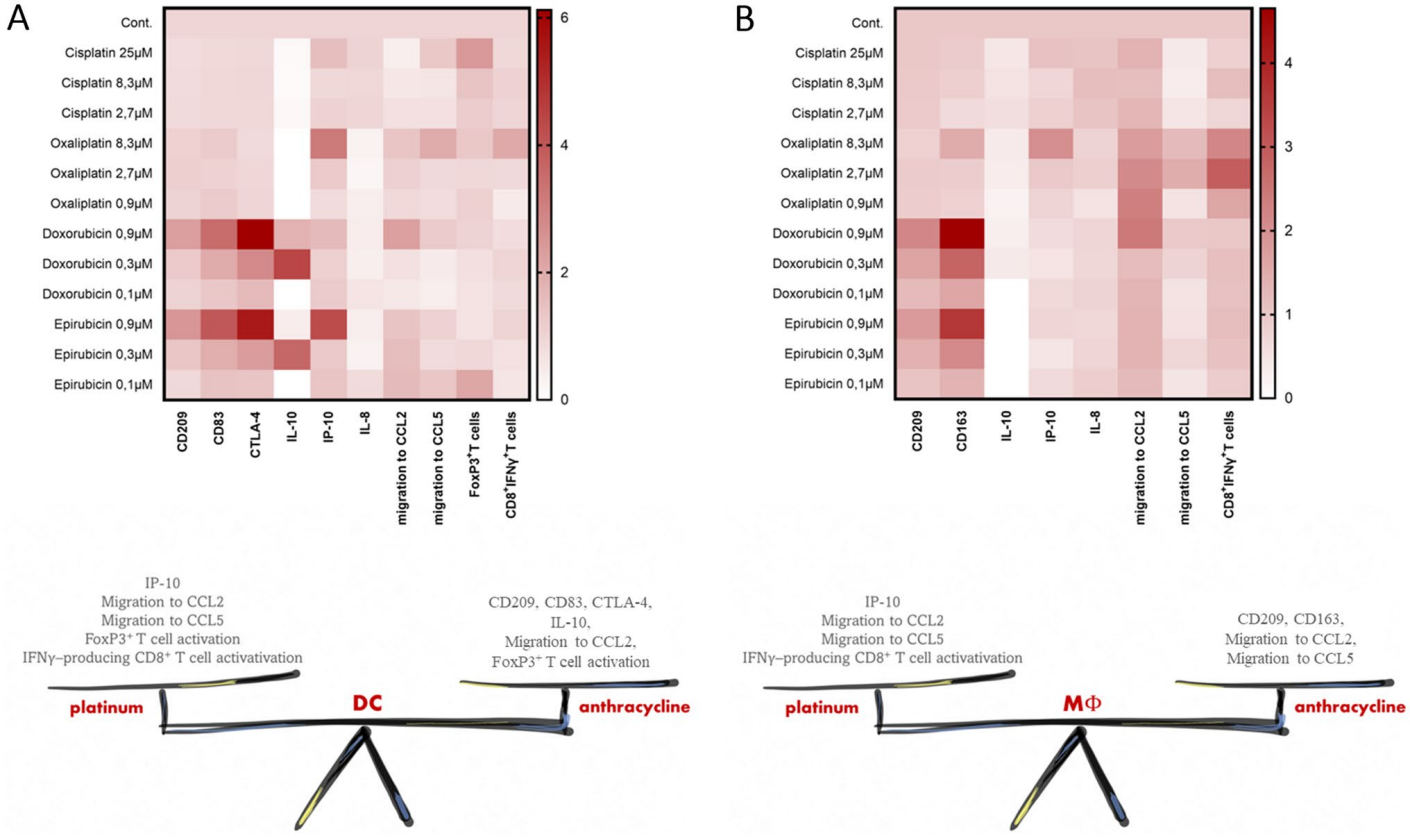
Platinums and anthracyclines in a concentration-dependent way differently alter the differentiation process and functions of both macrophages and dendritic cells. In order to better illustrate our observations we summarized our results in heat maps. Macrophages and dendritic cells are both sensitive to chemotherapeutic agents but the chemicals have different effects on APC differentiation. Additionally, platinums and anthracyclins differently alter the phenotypical and functional properties of dendritic cells and macrophages

The contribution of the largely immune cell-driven tumor microenvironment to tumor proliferation, survival, and spread is essential [14]. Antineoplastic chemotherapeutic agents that act on highly proliferating cells affect not only the viability of tumor cells, but also the functioning of immune cells. Plenty of effort has been made to evaluate and compare the efficacy and safety of oxaliplatin/cisplatin-based or doxorubicin/epirubicin-based treatments [15, 16]. Although the cytotoxic effects of these drugs on tumors are well known, their effects on the tumor microenvironment have been less studied [17, 18] and have been only exceptionally analyzed in comparative studies [19]. The different immunomodulatory potential of agents with the same mechanism of action should also be considered, as this may influence the therapeutic recommendation, rationalize the possibility of co-therapies, and may explain treatment failure. We investigated the immunomodulatory potential of two platinums and two anthracyclines, namely, oxaliplatin and cisplatin, doxorubicin, and epirubicin, respectively, on the differentiation of human monocyte-derived macrophages or DCs (Fig. 7) summarizes and compares the changes induced by chemotherapeutic agents in macrophages and DCs differentiated from monocytes in our experimental setup.

Several strategies have been developed to influence the balance between tumor-promoting and tumor-attacking subtypes of innate immune cells by “reprogramming” their differentiation. Targeted depletion of tolerogenic cells also offers a therapeutic option. Induction of cell death when using chemotherapy is certainly the main goal of therapeutic intervention, so monitoring the cell death susceptibility of plastic cell populations in TME may be clinically relevant. Some chemotherapeutic drugs, such as epirubicin, doxorubicin, oxaliplatin, may increase the immunogenicity of tumor cells in a dose- and schedule-dependent manner by activating immunogenic cell death (ICD) [20, 21]. Some studies have compared the sensitivity of M1 and M2 macrophages to cell death induced by different stimuli [7], but DC subpopulations have not yet been studied in this regard. To induce cell death in subpopulations of macrophages and DCs, very high doses of cisplatin and oxaliplatin were required, far exceeding the clinically achievable Cmax [11]. No significant difference was found in the susceptibility of immunogenic and tolerogenic cell populations to cell death after epirubicin treatment, but inflammatory M1 macrophages were more sensitive to doxorubicin-induced cell death than M2 macrophages. These results highlight that the differentiation of the two cell populations can influence their susceptibility to cell death, a more precise understanding of which requires further study.

These results highlight that the differentiation of the two cell populations can influence their susceptibility to cell death, a more precise understanding of which requires further study.

The most important effect of DCs in the regulation of anti-tumor immunity is the initiation of anti-tumor T-cell responses [22], whereas macrophages are critical components of TME [23]. Presumably, regulation of the immunogenicity of DCs is most critical in the early stages of tumor development, whereas, in advanced cancer, induction of the transition from anti-inflammatory macrophages to pro-inflammatory macrophages promises therapeutic success.

The most successful breakthrough in the field of tumor therapy has been the introduction of immune checkpoint inhibitors (ICI). However, these drugs do not work in a significant percentage of the population for reasons unknown so far. To make their impact as wide as possible, finding and introducing optimal co-therapies may be one of the solutions. Co-treatment with chemotherapy and ICI such as doxorubicin and the PD-1 inhibitor nivolumab may increase the overall survival rate [24]. Innate immune cells in the TME can significantly affect the success of ICI therapies, through their immunosuppressive ability, their critical effect on both naive and effector T-cell activation, or the expression of costimulatory molecules. The presence of anthracyclines during their differentiation strongly induced CTLA-4 expression on DCs, suggesting the potential success of anti-CTLA-4 co-therapy. Low-dose epirubicin-driven DC differentiation increased Treg but inhibited cytotoxic T-lymphocyte polarization, again raising the possibility of potential ICI co-therapy.

The use of chemotherapy may also worsen the prognosis by increasing the ability of the tumor metastasis. Innate immune cells and especially tumor-associated macrophages (TAM) affect the shift from the “noninvasive” tumor isoform to the “invasive” isoform by regulating angiogenesis, intravasation, and pre-metastatic niche formation. The CCL2-CCR2 signaling pathway promotes the early recruitment of inflammatory monocytes to the pre-metastatic niche. Here, the recruited monocytes differentiate into metastasis-associated macrophages (MAMs) [5, 6]*.* Accordingly, the blockade of CCL2 may prevent the development of cancer metastases [25]*.* However, inflammatory monocytes recruited by CCL2 can also differentiate into monocyte-derived dendritic cells, which can present antigens to CD8 + and CD4 + T cells, thus assisting and stimulating the adaptive anti-tumor immune response. Additionally, the CCL5-CCR5 axis supports cancer progression by stimulating immunosuppressive TAM polarization in TME, increasing tumor invasiveness, and metastasis formation [26]. To our surprise, strong intratypic differences were observed between both anthracyclines and platinums when their effects on macrophage chemotaxis were tested. Macrophages differentiated in the presence of cisplatin versus oxaliplatin and doxorubicin versus epirubicin have principally different migratory potentials (Fig. 6).

Epirubicin treatment during differentiation consistently increased the migration intensity of non-treated moDCs and DCs’s movement toward CCL2. Although there were sudden changes in the effects on DCs migration with increasing doses. High but still non-toxic doses of cisplatin, oxaliplatin, or doxorubicin treatments modified the propensity of DCs to move toward both CCL2 and CCL5. These results indicate that chemotherapy may modify the migration of monocyte-derived cell populations in a drug- and cell-type-specific manner and highlights the importance of further studies to elucidate the role of innate immune cells in metastasis following chemotherapy.

To optimize the result, the treatment should be tailored and the most effective concentrations of drugs acting on both tumor cells and immune cells should be calculated [3]. Based on our results, different aspects of the differentiation of monocytes into macrophages or DCs also appeared to be drug- and dose-dependent, affecting their functional characteristics.

Taken together, these results suggest that the optimal dose and exact schedule of chemotherapy, which may vary from tumor type to tumor type, are both critical determinants of immune outcome.

## Supplementary Information

Below is the link to the electronic supplementary material.

Supplementary Figure 1. Viability of macrophages and dendritic cells following cisplatin, oxaliplatin, doxorubicin or epirubicin-driven differentiation. CD14+ monocytes were cultured with 50 ng/ml recombinant M-CSF (for macrophages) or 100 ng/ml IL-4 and 80 ng/ml GM-CSF (for dendritic cells). Cisplatin, oxaliplatin, doxorubicin or epirubicin were added to freshly isolated monocytes at the indicated doses for five days. On day five, the viability of cells was measured by 7-aminoactinomycin D (7-AAD) staining using flow cytometry. Cell viability was also measured with Cell Counting Kit-8; CCK-8 solution was added to each well and then incubated for 4 hours. All experiments were performed in quadruplicate, and cell viability (%) was expressed as a percentage relative to the untreated control cells. In the statistical analysis, ANOVA followed by Bonferroni’s post hoc test was used for the comparison. The results were expressed as mean± standard deviation. Supplementary file1 (TIF 1431 KB)

Supplementary Figure 2. The presence of chemotherapeutic agents during differentiation does not modifiy the pattern of cell surface markers in macrophages. CD14+ monocytes were cultured with recombinant 50 ng/ml recombinant M-CSF. Cisplatin/oxaliplatin/doxorubicin/epirubicin was added to freshly isolated monocytes at the indicated doses for five days. On day five, the cell surface expression of CD14, HLA-DQ, CD86, CTLA-4 and PD-L1 were analyzed on monocyte-derived macrophages by flow cytometry. The MFI (median fluorescence intensity) were calculated from three independent experiments +SD at least. In the statistical analysis, ANOVA followed by Bonferroni’s post hoc test was used for the comparison. The results were expressed as mean± standard deviation. Supplementary file2 (TIF 1004 KB)

Supplementary Figure 3. The presence of chemotherapeutic agents during differentiation does not modifiy the pattern of cell surface markers in dendritic cells. CD14+ monocytes were cultured with recombinant 100 ng/ml IL-4 and 80 ng/ml GM-CSF. Cisplatin/oxaliplatin/doxorubicin/epirubicin was added to freshly isolated monocytes at the indicated doses for five days. On day five, the cell surface expression of CD206, CD80, PD-L1 and CD1a were analyzed on monocyte-derived DCs by flow cytometry. The MFI (median fluorescence intensity) and the mean values of the cells' ratio positive for the measured surface molecules were calculated from five independent experiments +SD at least. In the statistical analysis, ANOVA followed by Bonferroni’s post hoc test was used for the comparison. The results were expressed as mean± standard deviation. Supplementary file3 (TIF 846 KB)

Supplementary Figure 4. The presence of chemotherapeutic agents during differentiation does not modify the PMA-induced ROS production of macrophages. CD14+ monocytes were cultured with 50 ng/ml recombinant M-CSF (for macrophages) or 100 ng/ml IL-4 and 80 ng/ml GM-CSF (for dendritic cells). Cisplatin, oxaliplatin, doxorubicin or epirubicin were added to freshly isolated monocytes at the indicated doses for five days. On day five, the (A) macrophages or (B) DCs were stimulated with 100 nM phorbol 12‐myristate 13‐acetate (PMA). Following incubation times (0, 15, 30 mins and 1, 1.5, 2, 3, 4, 5, 6 hours), ROS production was measured in relative optical density (OD) at 630 nm using a microplate reader. The bars show the average density of three independent experiments +SD. Supplementary file4 (TIF 1221 KB)

Supplementary Figure 5. Gating strategy for T-cell-polarizing assay. CD14+ monocytes were cultured with 50 ng/ml recombinant M-CSF (for macrophages) or 100 ng/ml IL-4 and 80 ng/ml GM-CSF (for dendritic cells) in the presence of cisplatin/oxaliplatin/doxorubicin or epirubicin for five days. On day five macrophages or dendritic cells were co-cultured with allogenous peripheral blood lymphocytes (PBL) at a monocyte-derived cell: T-cell ratio of 1 : 10 at 37°C. After three, five, or nine days the T cells were stimulated with 1μg/ml ionomycin and 20 ng/ml phorbol-myristic acetate (PMA) for 4 hours, and the vesicular transport was inhibited. Panel A shows the size and granulation of T-cell cultures or T cell + APC cocultures. Panel B depicts the gating of CD8+ cytotoxic, and CD4+ helper T cells. The histogram of panel C shows the gating strategy of APC-labeled marker (IFNγ or FoxP3) positive cells. Panel D and E show the gating strategy of regulatory T cells. Panel E dot plots depict the further gating strategy of CD4+ T cells. Supplementary file5 (TIF 2359 KB)

## Data Availability

Data will be made available on reasonable request.
