# Supplementary figures and images for: Comparison of the immunomodulatory potential of platinum-based anti-cancer drugs and anthracyclins on human monocyte-derived cells
Source: Cancer Chemother Pharmacol. 2022 Nov 30;91(1):53–66. doi: 10.1007/s00280-022-04497-1 (PMC9813091; doi:10.1007/s00280-022-04497-1)

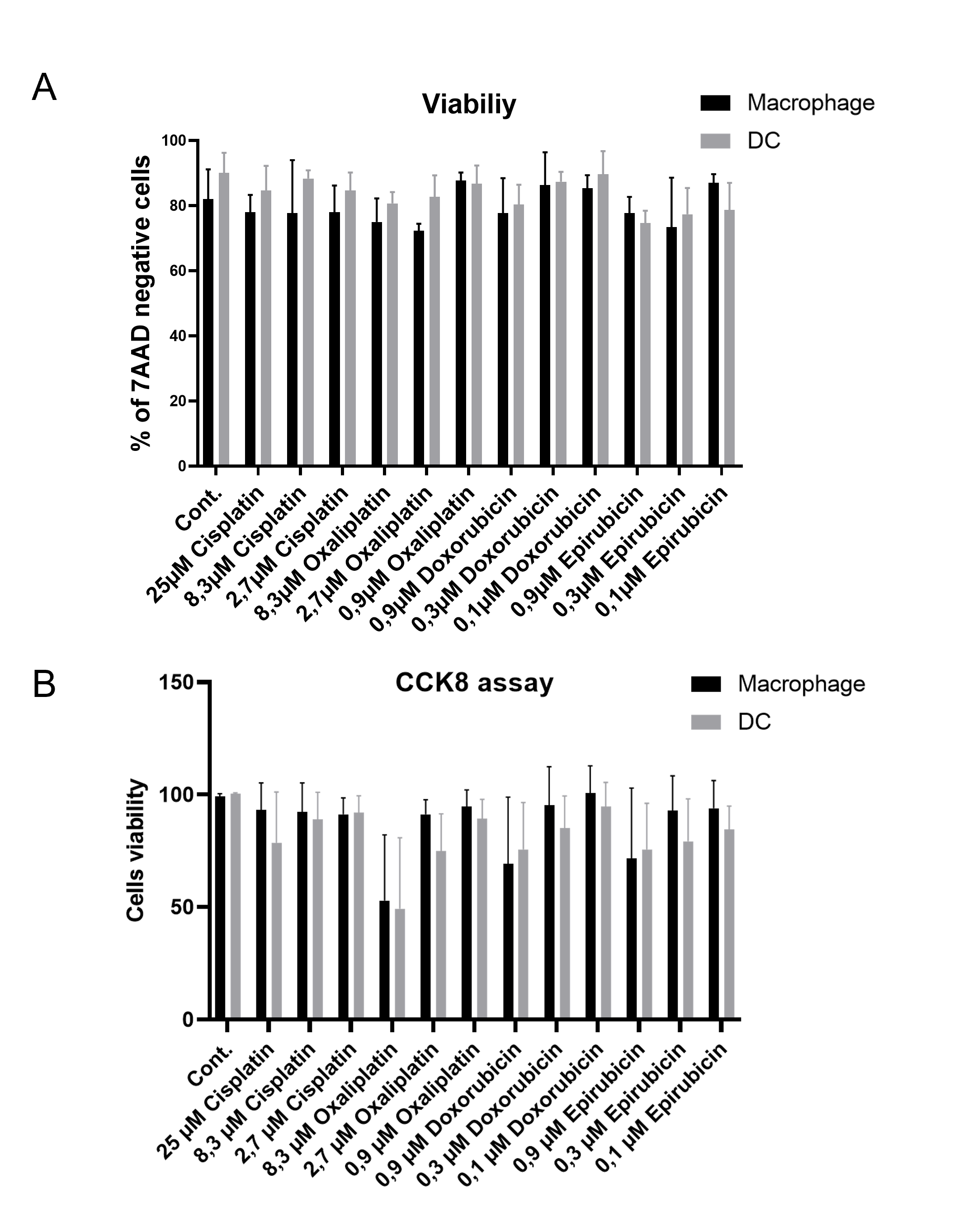

Supplement: Supplementary file 1 — Supplementary Figure 1. Viability of macrophages and dendritic cells following cisplatin, oxaliplatin, doxorubicin or epirubicin-driven differentiation. CD14+ monocytes were cultured with 50 ng/ml recombinant M-CSF (for macrophages) or 100 ng/ml IL-4 and 80 ng/ml GM-CSF (for dendritic cells). Cisplatin, oxaliplatin, doxorubicin or epirubicin were added to freshly isolated monocytes at the indicated doses for five days. On day five, the viability of cells was measured by 7-aminoactinomycin D (7-AAD) staining using flow cytometry. Cell viability was also measured with Cell Counting Kit-8; CCK-8 solution was added to each well and then incubated for 4 hours. All experiments were performed in quadruplicate, and cell viability (%) was expressed as a percentage relative to the untreated control cells. In the statistical analysis, ANOVA followed by Bonferroni’s post hoc test was used for the comparison. The results were expressed as mean± standard deviation. Supplementary file1 (TIF 1431 KB) [file 280_2022_4497_MOESM1_ESM.tif]

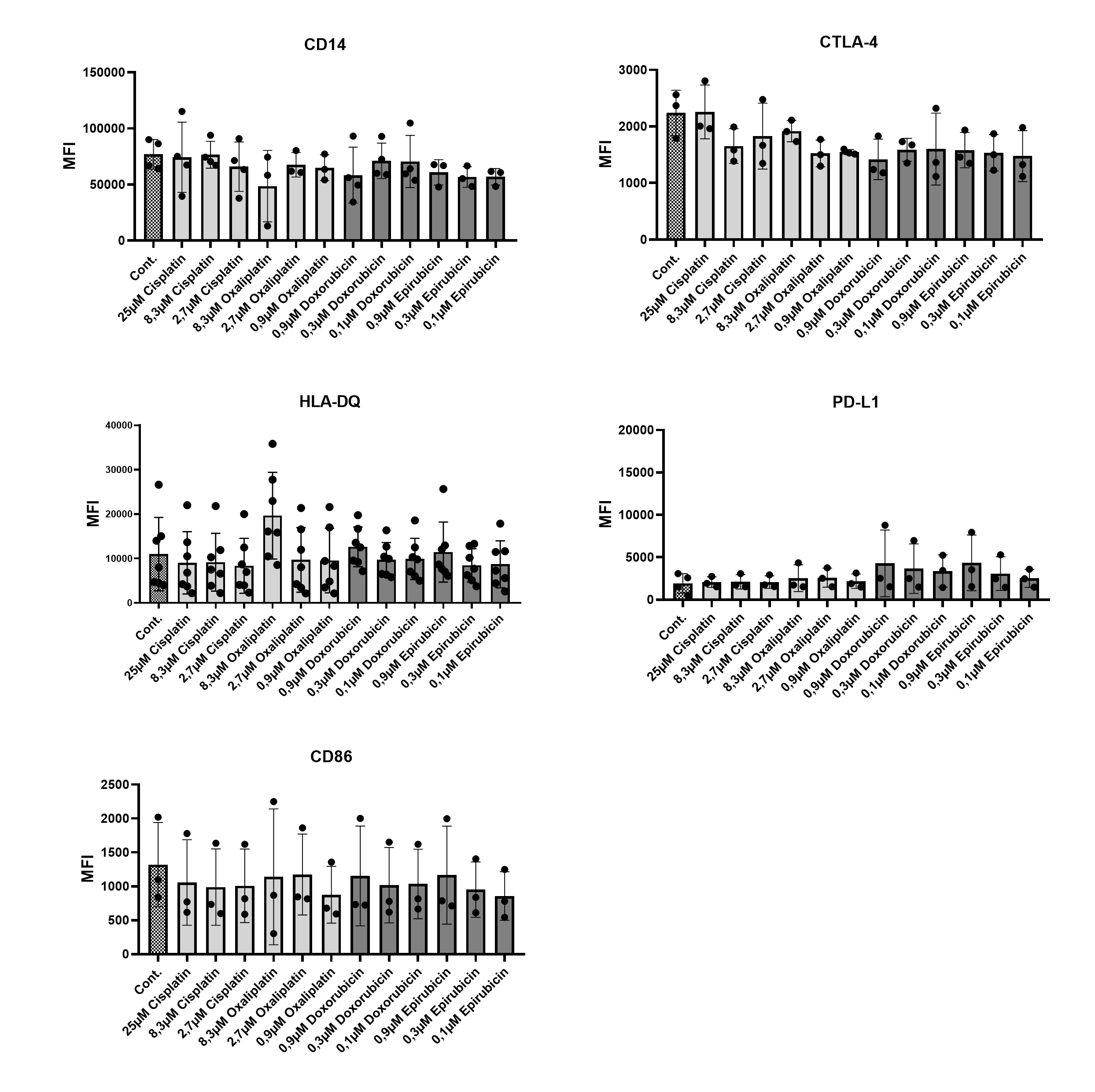

Supplement: Supplementary file 2 — Supplementary Figure 2. The presence of chemotherapeutic agents during differentiation does not modifiy the pattern of cell surface markers in macrophages. CD14+ monocytes were cultured with recombinant 50 ng/ml recombinant M-CSF. Cisplatin/oxaliplatin/doxorubicin/epirubicin was added to freshly isolated monocytes at the indicated doses for five days. On day five, the cell surface expression of CD14, HLA-DQ, CD86, CTLA-4 and PD-L1 were analyzed on monocyte-derived macrophages by flow cytometry. The MFI (median fluorescence intensity) were calculated from three independent experiments +SD at least. In the statistical analysis, ANOVA followed by Bonferroni’s post hoc test was used for the comparison. The results were expressed as mean± standard deviation. Supplementary file2 (TIF 1004 KB) [file 280_2022_4497_MOESM2_ESM.tif]

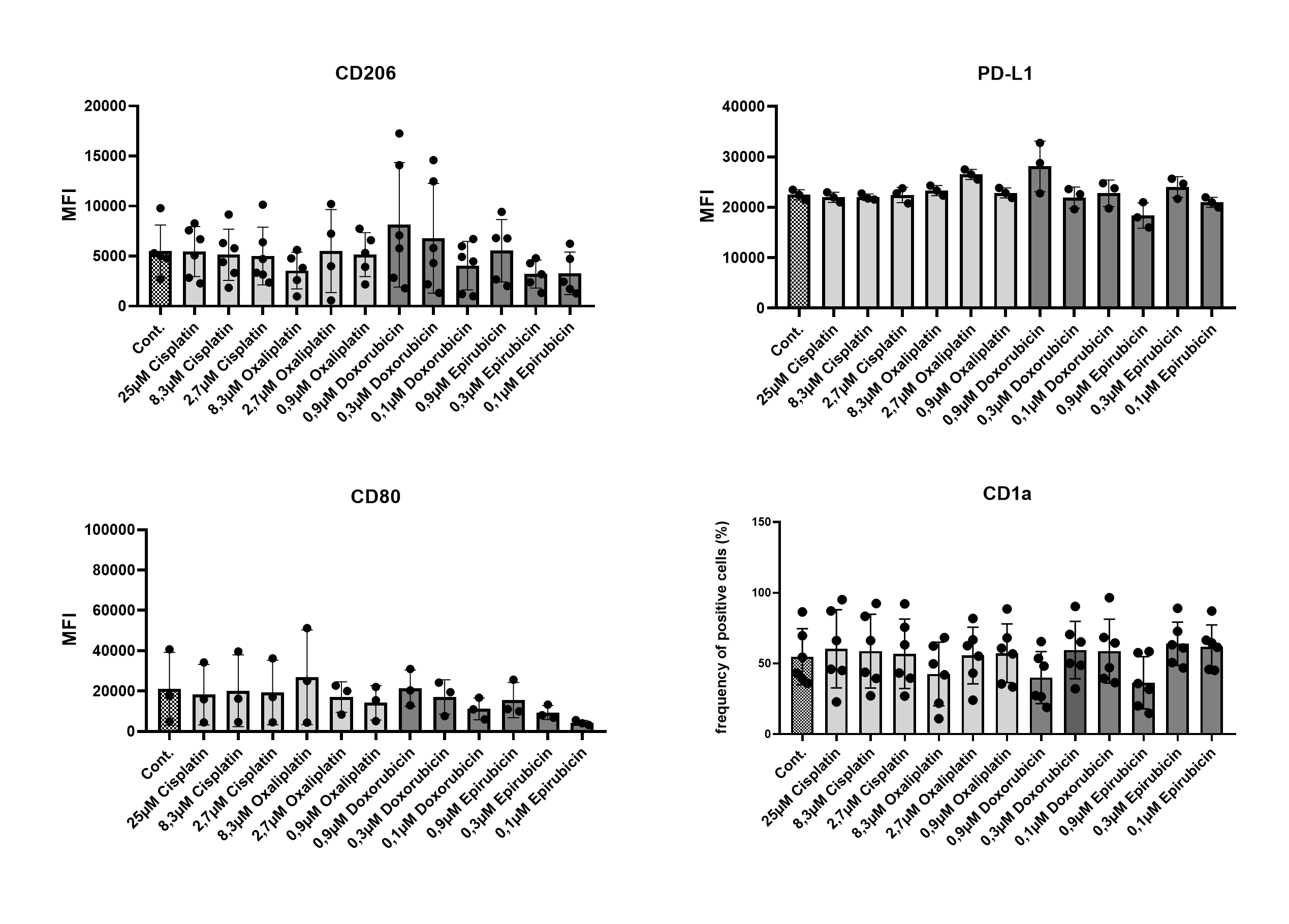

Supplement: Supplementary file 3 — Supplementary Figure 3. The presence of chemotherapeutic agents during differentiation does not modifiy the pattern of cell surface markers in dendritic cells. CD14+ monocytes were cultured with recombinant 100 ng/ml IL-4 and 80 ng/ml GM-CSF. Cisplatin/oxaliplatin/doxorubicin/epirubicin was added to freshly isolated monocytes at the indicated doses for five days. On day five, the cell surface expression of CD206, CD80, PD-L1 and CD1a were analyzed on monocyte-derived DCs by flow cytometry. The MFI (median fluorescence intensity) and the mean values of the cells' ratio positive for the measured surface molecules were calculated from five independent experiments +SD at least. In the statistical analysis, ANOVA followed by Bonferroni’s post hoc test was used for the comparison. The results were expressed as mean± standard deviation. Supplementary file3 (TIF 846 KB) [file 280_2022_4497_MOESM3_ESM.tif]

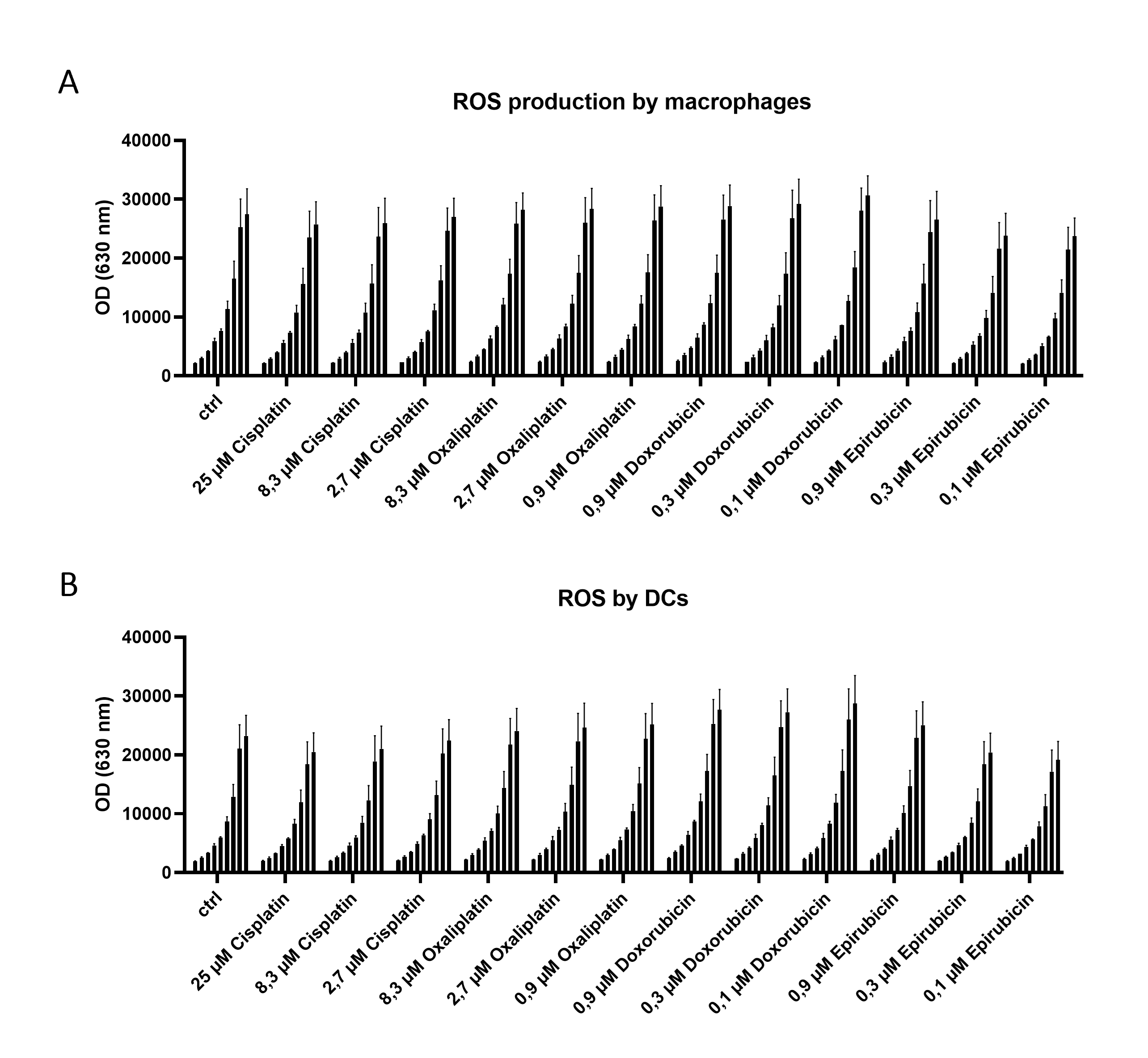

Supplement: Supplementary file 4 — Supplementary Figure 4. The presence of chemotherapeutic agents during differentiation does not modify the PMA-induced ROS production of macrophages. CD14+ monocytes were cultured with 50 ng/ml recombinant M-CSF (for macrophages) or 100 ng/ml IL-4 and 80 ng/ml GM-CSF (for dendritic cells). Cisplatin, oxaliplatin, doxorubicin or epirubicin were added to freshly isolated monocytes at the indicated doses for five days. On day five, the (A) macrophages or (B) DCs were stimulated with 100 nM phorbol 12‐myristate 13‐acetate (PMA). Following incubation times (0, 15, 30 mins and 1, 1.5, 2, 3, 4, 5, 6 hours), ROS production was measured in relative optical density (OD) at 630 nm using a microplate reader. The bars show the average density of three independent experiments +SD. Supplementary file4 (TIF 1221 KB) [file 280_2022_4497_MOESM4_ESM.tif]

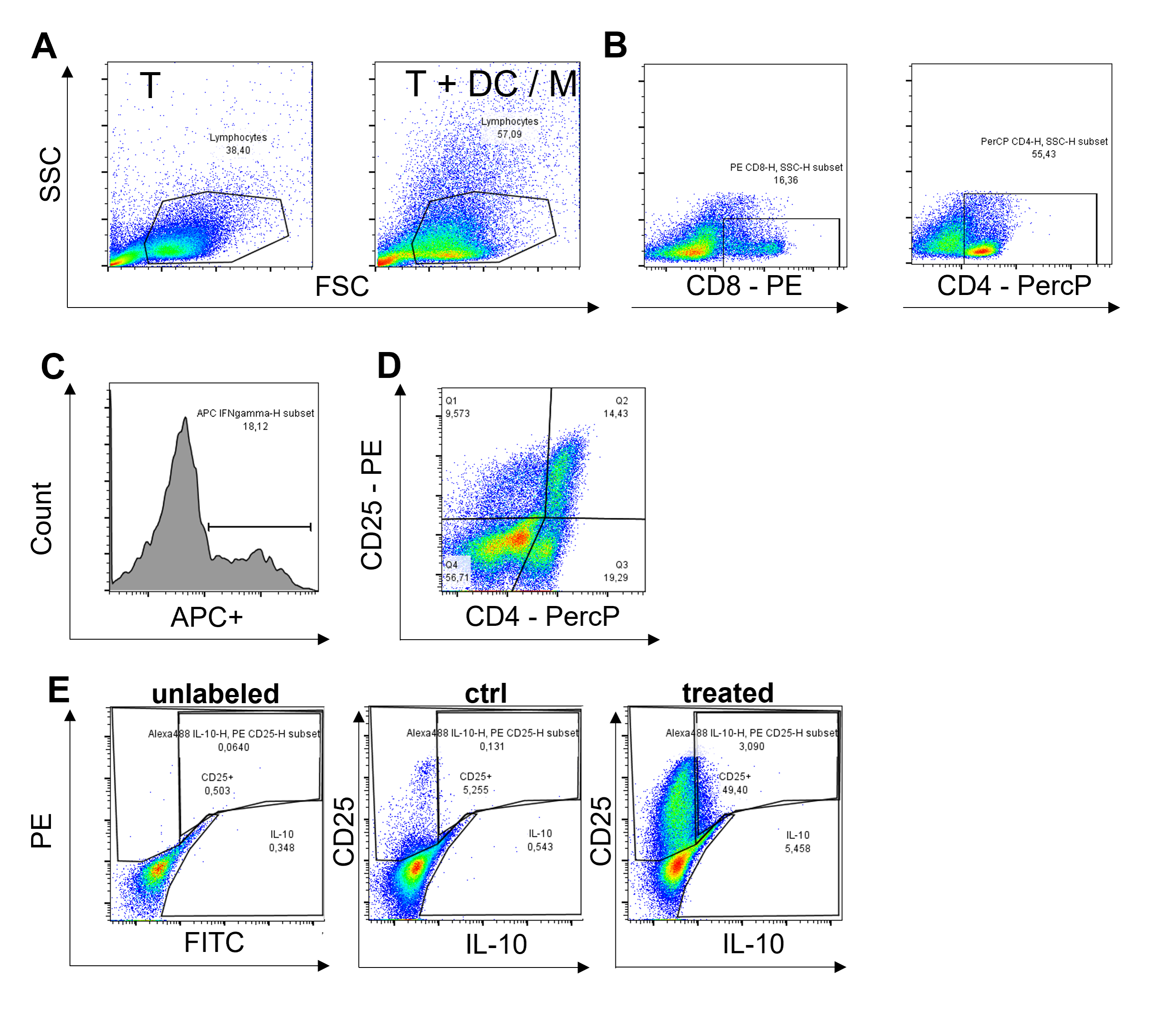

Supplement: Supplementary file 5 — Supplementary Figure 5. Gating strategy for T-cell-polarizing assay. CD14+ monocytes were cultured with 50 ng/ml recombinant M-CSF (for macrophages) or 100 ng/ml IL-4 and 80 ng/ml GM-CSF (for dendritic cells) in the presence of cisplatin/oxaliplatin/doxorubicin or epirubicin for five days. On day five macrophages or dendritic cells were co-cultured with allogenous peripheral blood lymphocytes (PBL) at a monocyte-derived cell: T-cell ratio of 1 : 10 at 37°C. After three, five, or nine days the T cells were stimulated with 1μg/ml ionomycin and 20 ng/ml phorbol-myristic acetate (PMA) for 4 hours, and the vesicular transport was inhibited. Panel A shows the size and granulation of T-cell cultures or T cell + APC cocultures. Panel B depicts the gating of CD8+ cytotoxic, and CD4+ helper T cells. The histogram of panel C shows the gating strategy of APC-labeled marker (IFNγ or FoxP3) positive cells. Panel D and E show the gating strategy of regulatory T cells. Panel E dot plots depict the further gating strategy of CD4+ T cells. Supplementary file5 (TIF 2359 KB) [file 280_2022_4497_MOESM5_ESM.tif]
